# Supplementary material for: Epigenomics Analysis of the Suppression Role of SIRT1 via H3K9 Deacetylation in Preadipocyte Differentiation
Source: Int J Mol Sci. 2023 Jul 10;24(14):11281. doi: 10.3390/ijms241411281 (PMC10379189; doi:10.3390/ijms241411281)
Supplement: Supplementary file 1 [file ijms-24-11281-s001.zip › ijms-2469483-supplementary.docx]

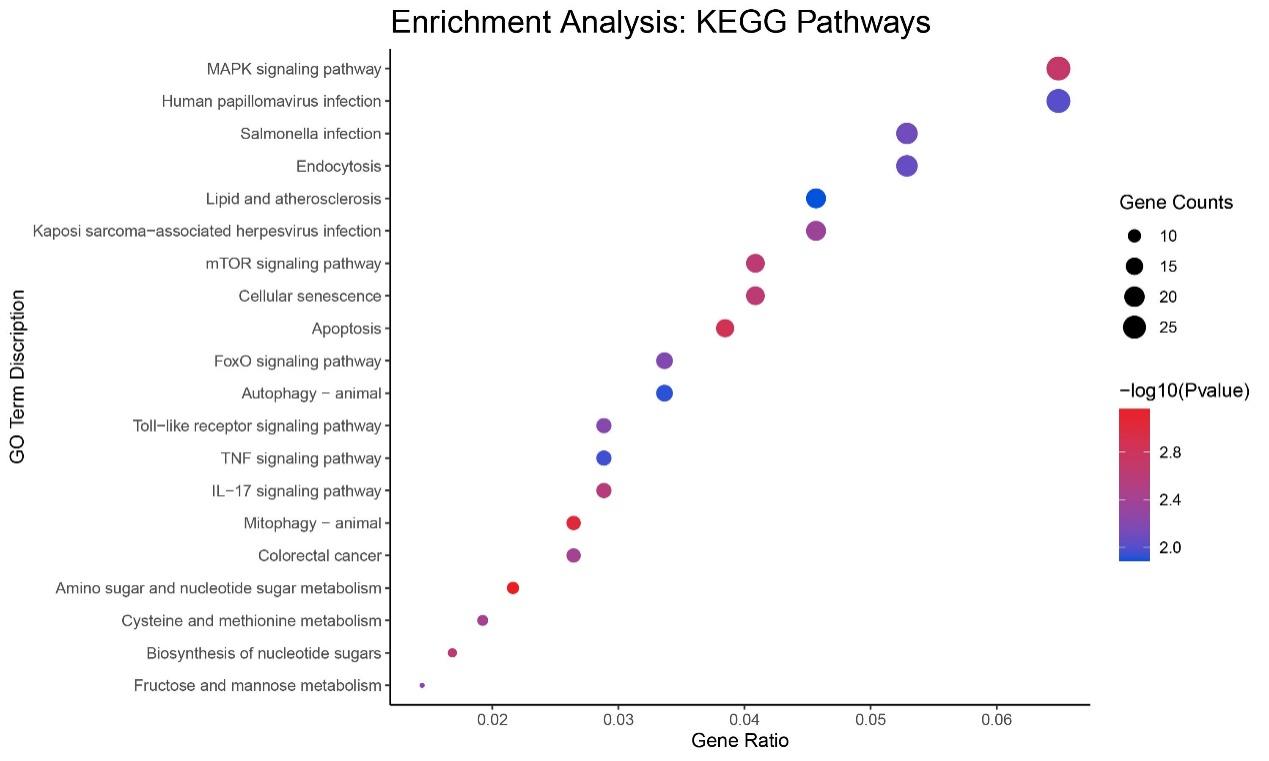


**Figure S1.** KEGG enrichment pathway analysis of overlap genes in ChIP-seq and mRNA-seq.

**Table S1.** The shRNA sequence of *SIRT1* and the negative control.

| **Name** | **shRNA sequence（5’-3’）** |
| --- | --- |
| *SIRT1*-shRNA | F:GATCCGAGCTTCTTGGAGACTGTGACGTAATTCAAGAGATTACGTCACAGTCTCCAAGAAGCTCTTTTTTG |
|  | R:AATTCAAAAAAGAGCTTCTTGGAGACTGTGACGTAATCTCTTGAATTACGTCACAGTCTCCAAGAAGCTCG |
| NC-shRNA | F:GATCCGTTCTCCGAACGTGTCACGTAATTCAAGAGATTACGTGACACGTTCGGAGAATTTTTTC |
|  | R:AATTGAAAAAATTCTCCGAACGTGTCACGTAATCTCTTGAATTACGTGACACGTTCGGAGAACG |

**Table S2.** The sequence of siRNA.

| **siRNA Number** | **Sense (5’-3’)** | **Antisense (5’-3’)** |
| --- | --- | --- |
| si*TRIM23* | GCUUCUUAGUCUCCAUAAATT | UUUAUGGAGACUAAGAAGCTT |
| Negative Control | UUCUCCGAACGUGUCACGUTT | ACGUGACACGUUCGGAGAATT |

**Table S3.** Primers used in RT-qPCR.

| **Gene Name** | **Primer Sequence (5′ to 3′)** |
| --- | --- |
| SIRT1 | F: TGGGGTTTCTGTTTCTTGTGG  R: CTTGAGGATCTGGAAGGTCTGG |
| GAPDH | F: CCACGAGAAGTATAACAACACC  R: GTCATAAGTCCCTCCACGAT |
| PPARγ | F: CGTGGACCTTTCTATGATGGA  R: GCTCTTGGGAACGGAATG |
| PGC-1a | F：GTACCAGCACGAAAGGCTCAA  R：ATCACACGGCGCTCTTCAA |
| C/EBPα | F: GTGGACAAGAACAGCAACGAGTA  R: GCGGTCATTGTCACTGGTCAG |
| AP2 | F: GGAAAGTCAAGAGCATCGTAAAC  R: CACCATCTTATCATCCACGAGTT |
| LPL | F：ACACAGCTGAGGACACTTGCC  R：GCCATGGATCACCACAAAGG |
| ATGL | F: TGCGCAACCTCTACCGCCTC  R: AAGGGGTTGGGCCGGTTCAG |
| HSL | F: GCTGGGCCGTCAAGCACTGC  R: GTGGCCGGGTAGGCTGCCAT |
| CPT1A | F：CATCCAGGCGGCAAGAG  R：GAGCAGAGCGGAATCGT |
| PTEN | F: GGAAAGGGACGAACTGGTGTAA  R: TGTCTCTGGTCCTTACTTCCCC |
| FOXO1 | F：CCGCATCCATGGACAACAAC  R：CCGTCATGATGGGAGAGAGC |
| SREBP1 | F：GCATCAACCACGGTCCCA  R：AGCACCAGCAGCCCATTC |
| SCAP | F：CCATGTGCACTTCAAGGAGGA  R：ATGTCGATCTTGCGTGTGGAG |
| FASN | F：ACCTCGTGAAGGCTGTGACTCA  R：TGAGTCGAGGCCAAGGTCTGAA |
| ACACA | F：CATCTTGTCCGAAACGTCGAT  R：CCCTTCGAACATACACCTCCA |
| SCD | F：AGTCACCGAACCTACAAAG  R：ACAAGCAGCCAACCCAC |
| DGAT2 | F:CATGTACACATTCTGCACCGATT  R:TGACCTCCTGCCACCTTTCT |
| FABP3 | F：GAACTCGACTCCCAGCTTGAA  R：AAGCCTACCACAATCATCGAAG |
| PLIN2 | F：TGGTCTCCTCGGCTTACATCA  R：TCATGCCCTTCTCTGCCATC |
| CCNB1 | F:AATCGGTGACTTTGCCTTTG  R:AGGGCGACCCAGACTAAAAT |
| CCND1 | F:GATCAGATGTGACCCGGACT  R:TCCTCCTCCTCTTCCTCCTC |
| CCNE1 | F:GTGGCATTTAAGTCCCCTGA  R:AGGATACTGAGGCAGGAGCA |
| KI67 | F:AGAGCCAACACTCCGAAGAA  R:GCTGAGCTTTCCCTATGACG |

**Table S4.** Primers used in ChIP-qPCR.

| **Gene Name** | **Primer Sequence (5′ to 3′)** |
| --- | --- |
| FOXO1 | F: TCTGGGGGCTCTAGAAGAGA  R: CGTCAACCCACTACTGTTGG |
| HIF1A | F: GCAGTGGTACCTCCACGAAA  R: TGAGCAGACCAGTCTTAGGC |
| PPARGC1A | F: CTGGGATGGTGCTTCACTAA  R: TTCTCTGGGGCAGCTTATCT |
| UCP2 | F: GCCCCTGTTTGAGTACCTGT  R: ATCCCACCCCTACCTTCTCT |

**Table S5.** GO enrichments of downregulated genes from ChIP-seq results.

| **GO** | **Description** | ***P* value** |
| --- | --- | --- |
| GO:0048518 | positive regulation of biological process | 1.50E-08 |
| GO:0140110 | transcription regulator activity | 1.71E-06 |
| GO:0010604 | positive regulation of macromolecule metabolic process | 2.25E-06 |
| GO:0009893 | positive regulation of metabolic process | 4.15E-06 |
| GO:0001228 | DNA-binding transcription activator activity, RNA polymerase II-specific | 6.44E-06 |
| GO:0031325 | positive regulation of cellular metabolic process | 1.03E-05 |
| GO:0010468 | regulation of gene expression | 5.89E-05 |
| GO:0006357 | regulation of transcription by RNA polymerase II | 0.00015373 |
| GO:0051254 | positive regulation of RNA metabolic process | 0.00021174 |
| GO:0033993 | response to lipid | 0.00022029 |
| GO:0045598 | regulation of fat cell differentiation | 0.00024876 |

**Table S6.** ChIP-seq analysis of the KEGG lipid metabolism-related pathway of peak down-regulated genes.

| **Term** | **pvalue** |
| --- | --- |
| PI3K-Akt signaling pathway | 0.042050449 |
| Insulin resistance | 0.053464083 |
| HIF-1 signaling pathway | 0.058525586 |
| Fatty acid biosynthesis | 0.095124377 |
